# Application of the novel and convenient IR/MAR gene amplification technology to the production of recombinant protein pharmaceuticals

**DOI:** 10.1186/1753-6561-5-S8-P131

**Published:** 2011-11-22

**Authors:** Yoshio Araki, Chiemi Noguchi, Tetsuro Hamafuji, Hiroshi Nose, Daisuke Miki, Noriaki Shimizu

**Affiliations:** 1 Graduate School of Biosphere Science, Hiroshima University, Higashi-hiroshima, 739-8521, Japan; 2 Transgenic Inc., Kobe, 650-0047, Japan; 3 Tosoh Co., Tokyo, 252-1123, Japan

## 

Amplification of DHFR gene in CHO cells by selection of MTx has been widely applied to the establishment of stable cell lines that efficiently produce recombinant protein pharmaceuticals. However, the DHFR/MTx technology was highly time-and labor-consuming. On the other hand, we had found that a plasmid bearing a mammalian replication initiation region (IR) and a matrix attachment region (MAR) initiates gene amplification in mammalian cells, and it quite efficiently generate the chromosomal HSR and/or the extrachromosomal DMs [1,2]. This is a completely original technology of gene amplification, and we have revealed the mechanism why and how such plasmid may mimic gene amplification [2,3]. Now, we aimed to adopt this technology to the industrial production of recombinant protein pharmaceuticals.

We constructed plasmids with or without IR/MAR, with several promoters for drug resistant gene, antibody gene or Fc receptor gene, and with various orientations of these elements. The plasmid DNA with several physical structures were transfected to human COLO 320DM or hamster CHO DG44 cells, with or without another DNA. The transformed cells were selected by various conditions. The polyclonal transformants or the cloned cells were evaluated by the protein production (ELISA), as well as by the structure of amplified region (FISH).

**Figure 1:**
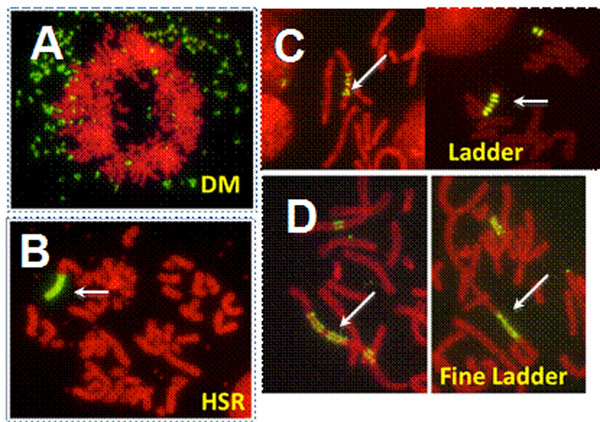
The IR/MAR plasmid can generates DMs (A), HSR (B), Ladder-HSR (C), and Fine ladder-HSR (D). Among the metaphase chromosome spread, we detected the plasmid sequence by FISH in green. Gene expression was generally higher from DMs than HSR. However, DMs are usually hard to be generated in CHO cells. The IR/MAR plasmid can efficiently generate the ladder and the fine ladder structure that is active in transcription.

As a result, the usage of IR/MAR technology enabled us to obtain cells, in which the introduced genes were amplified to a few hundreds to thousands copies per cells as DMs or HSR of various size and shape (Figure 1), which depended both on the vector constructs and the host cell lines. Such stable cells with amplified genes could be obtained within one month, and the protein production was increased more than a hundred-fold compared with the case without IR/MAR. A cell clone showed the specific production rate that reached almost the highest reported for antibody protein (45 pg/cell/day). Furthermore, we have found several novel ways that further improve the protein production level. For example, the combination of the IR/MAR and the DHFR/MTx technologies synergistically work and far more rapidly and easily generate the cells of higher production rate than previously.

In conclusion, the IR/MAR technology is a novel highly-competitive technology for use in recombinant protein production, and it further has potentials for improvement.
